# Harnessing magnetism: evaluation of safety, tolerance and feasibility of magnetic kidney stone retrieval in vivo in porcine models

**DOI:** 10.1007/s00240-024-01684-y

**Published:** 2024-12-24

**Authors:** Thomas Amiel, Shyam Srinivasan, Chiara Turrina, Florian Ebel, Michael Straub, Sebastian P. Schwaminger

**Affiliations:** 1 Department of Urology, University Hospital Klinikum Rechts der Isar, Technical University of Munich, Ismaninger Str. 22, 81675 Munich, Germany; 2 Chair of Bioseparation Engineering, School of Engineering and Design, Technical University of Munich (TUM), Boltzmannstr. 15, 85748 Garching, Germany; 3 Division of Medicinal Chemistry, Otto Loewi Research Center, Medical University of Graz, Neue Stiftingtalstr. 6, 8010 Graz, Austria; 4 BioTechMed-Graz, Mozartgasse 12, 8010 Graz, Austria

**Keywords:** Kidney stone removal, Magnetic particles, Magnetic extraction, Calculi, Porcine model, Lithotripsy

## Abstract

The primary objective of urolithiasis therapy is complete stone removal and highest stone-clearance rates possible to minimize recurrence. A novel approach that employs a magnetic suspension and a magnetic probe for the passive collection and removal of small residual fragments was developed. This study assessed the feasibility of this system in porcine models. Five female domestic pigs underwent retrograde intrarenal surgery under general anaesthesia to assess the new magnetic system. Pre-analysed human calculi were endoscopically inserted and comminuted using lithotripsy. The magnetic suspension was applied, and the magnetic-stone fragment complex was extracted. After nephrectomy, independent blinded pathologists evaluated all the kidneys. Safety and tolerance assessments revealed no adverse events (i.e. no complications on the Clavien-Dindo scale > 1) or complications associated with treatment. This study revealed superficial urothelial damage in all animals, characterized by desquamation and inflammation, caused primarily by the insertion of access sheaths and laser lithotripsy. Residual magnetic particles were observed in the renal pelvis but did not show signs of toxicity even though this study is limited to the acute treatment. No pathological indicators were observed in the hemogram and urinalysis. Overall, the treatment did not cause any significant pathological changes. Preclinical in vivo evaluation of magnetic extraction of small rest fragments in porcine kidneys presents a promising, atraumatic approach for fragments removal. It demonstrated safety, tolerance, and feasibility that warrants clinical investigation. This method has the potential to increase stone-clearance rates with shorter extraction times, offering a possibility for addressing the challenge of urolithiasis in clinical practice.

## Introduction

Stone residue in the kidney after lithotripsy is a significant factor contributing to stone recurrence [1]. In the past, clinically insignificant rest fragments (CIRF) were left as residue to be expulsed by the patient post-surgery [2]. To date, there is no exact definition of CIRFs but there definition varies between fragments smaller than 1–5 mm [3]. EAU guidelines distinguish between residual fragments larger and smaller than 4 mm for follow-up of urology stones [4]. Nowadays the discussion of leaving residual fragments (RF) is more present than ever [5–7]. While new laser technologies have made dusting more efficient, even with large calculi, the issue of RF remains unsolved [5, 8]. RFs can serve as nuclei for secondary stone formation, leading to renal colic and increased re-treatment rates. Epidemiological data reveals re-treatment rates ranging from 20 to 60% when RFs persist after shockwave lithotripsy, retrograde intrarenal surgery and percutaneous nephrolithotomy (PNL) procedures [9, 10].

Laser-lithotripsy remains the gold standard for endoscopic stone disintegration, primarily used in flexible ureteroscopy (fURS) and PNL [11–13]. While PNL focuses on fragmentation and extraction through fluid evacuation, fURS prioritizes stone disintegration into dust, an established technique known as “Dusting” [14]. Despite being minimally invasive, stone dusting can prolong surgery durations and leave dust behind to be expulsed by the patient, which can persist and remain in the calyces [15]. Dust is not distinctly defined but usually refers to CIRFs derived from URS treatment [4].

The removal of RF remains a challenge due to the size and location of the kidney. Mechanical extraction using retrieval baskets is the most common method, but unable to guarantee the removal of all fragments, especially small fragments < 2 mm [2, 16, 17]. Moreover, suction approaches try to overcome the issues of extracting small fractals but often show issues with pressure handdling. [18, 19] Magnetic extraction is being considered to prevent CIRFs and even extract the tiniest fragments [20–23]. Magnetic extraction, originating from practices in the mineral industry and subsequently applied in various fields, particularly those based on iron oxide, has found application in biomedical fields such as medical imaging and cancer treatment [24].

Notably, this study introduces the first in vivo use of bare iron oxide particles for kidney stone removal, aiming to gain insight into the adsorption mechanism and the influencing parameters.

**Fig. 1:**
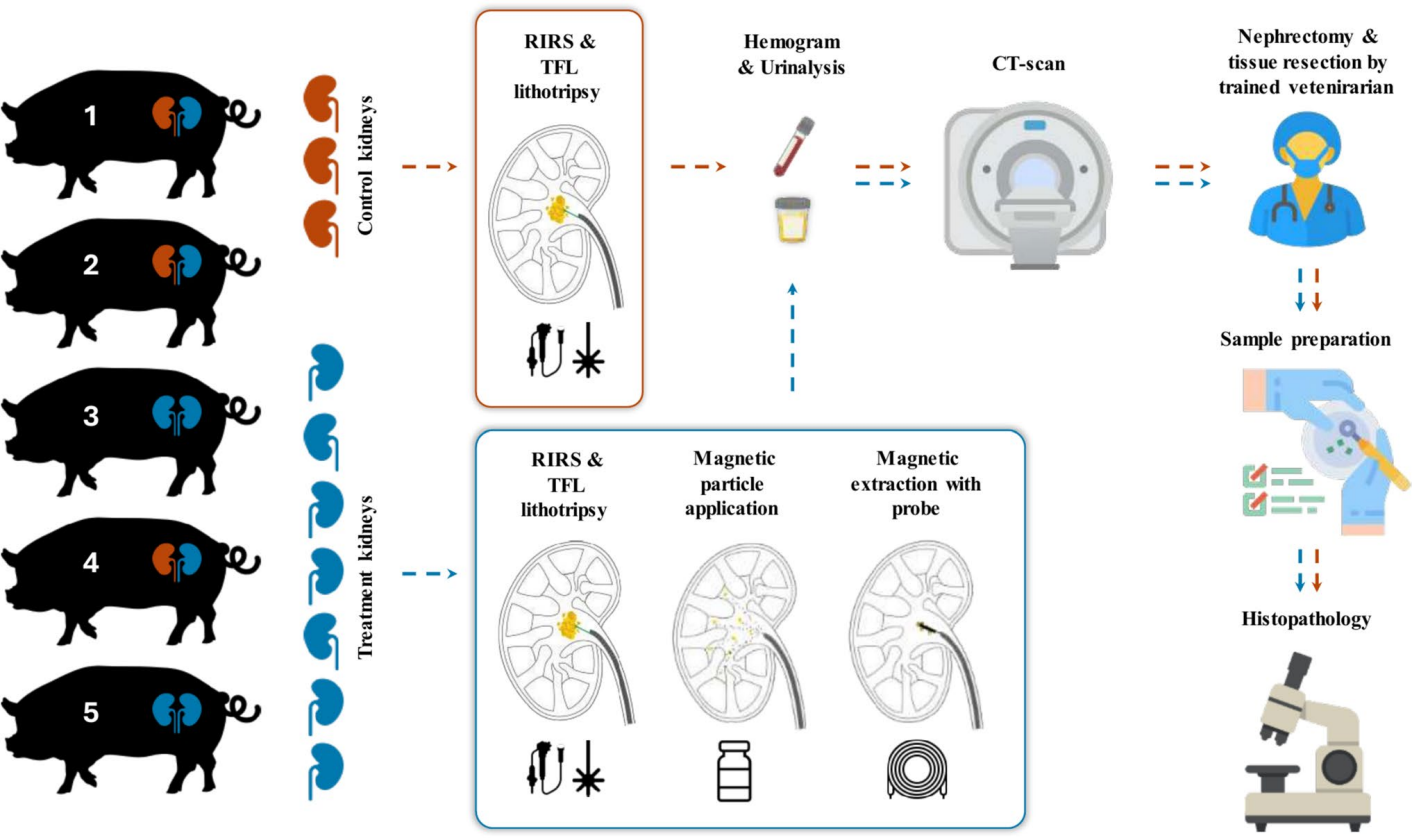
Schematic plan of the study (Icons made by iconmas, Freepik, Ylivdesign, pictogramer, Eucalyp, AmethystDesign, HANIS, ultimatearm from www.flaticon.com)

To date, different solutions are being developed, but none has achieved a consistent stone-free rate (SFR) after endoscopic lithotripsy. To address this challenge, we developed a novel technology, an extraction method based on the passive collection of magnetized stone dust using magnetophoretic attraction [22]. The system is a combination of magnetic particles (MP) that bind selectively to biomineral surfaces. After the fluid is applied to the kidney, the RF becomes magnetic and can be extracted with a magnetic probe. Here, we present the first preclinical evaluation of this novel product in an in vivo porcine kidney (Fig. 1).

## Materials and methods

An exploratory in vivo trial in five female pigs (59–69 kg) of sterile bare iron oxide particles [25], 2 mL 25 g/L, suspended in deionized water and a magnetic probe was conducted to evaluate safety endpoints (acute systemic toxicity, side effects and adverse events). 4 mL 0.9% sterile isotonic saline solution per mL of particles was used to reconstitute or “activate” the particles, which begins the process of agglomeration. A magnetic probe with 9 Fr NdFeB magnets are used for the extraction.

*Study Endpoints*: The primary endpoints of this study were to assess the safety and tolerance of the magnetic particle suspension and magnetic probe during Retrograde Intrarenal Surgery (RIRS) in an animal model. Safety evaluation focused on the potential occurrence of adverse events, including complications such as haemorrhage, hypotension, or any toxic/anaphylactic shock syndrome associated with the application of magnetic particles during surgery. The absence of any adverse events or complications would be indicative of positive safety and tolerance results. Toxicity assessment was based on histological and biological (urinalysis and hemogram) findings in the two groups, and the absence of significant differences would indicate that the application of magnetic particles and the use of magnetic probe were non-toxic.

*Animal Model*: Pigs were chosen due to the anatomical similarity of their kidneys to human kidneys. The volume of the porcine kidney pelvis is much smaller than in humans and the blood of pigs is, compared to humans, hypercoagulable [26]. Despite the differences, the clinical risks, including mechanical damage to the urothelium as well as signs of sensitivity, are transferable from pigs to humans. The considerations and setup of this study were in accordance with Good Laboratory Practices (GLPs), closely adhering to guidelines in ISO 10993-2:2006 for animal welfare requirements and ISO 10993-11:2017 for acute systemic toxicity. All procedures were carried out under general anaesthesia.

The study involved ten kidneys, with three pigs having intracorporeal control on one side and receiving treatment with the magnetic extraction method on the other side. The sample size of ten kidneys is defined by the endpoints which are acute complications during the acute treatment. Two pigs were treated bilaterally with the magnetic extraction method to attain sufficient samples for histopathology. All kidneys but two received a lithotripsy, where heterogenous small residual fragments (SRF)s were directly implanted. The size of these residual fragments varied between 3 to 20 mm. The fragments were prepared to fit through a 10/12 Fr ureteral access sheath (UAS) (ReTrace®, Coloplast, Denmark). Urine and blood samples were taken from each model before and after the surgery. Following the procedures, the animals were euthanized, and a high-dose CT scan with 0.7 mm section thickness was performed to assess the remaining fragments within the kidney’s collecting system. Subsequently, a bilateral nephrectomy was performed to evaluate the impact of the magnetic particle suspension and the magnetic probe on the kidney tissue by histopathological analysis. The nephrectomy procedure was conducted by a certified veterinarian. The blinded clinical pathology, i.e. hemogram and urinalysis, by *IMD Labor Oberland* (Am Kleistpark 1, 15,230 Frankfurt (Oder), Germany) and *SYNLAB vet. Labor Berlin* (Am Borsigturm 42, 13,507 Berlin, Germany) respectively.

*Control RIRS*: Standard RIRS procedures abiding international guidelines for kidney stone management were performed with the pigs in the dorsal lithotomy position [4, 27]. A cystoscopy was carried out to identify the ureteral orifices. Two hydrophilic guidewires (HiWire®, Cook Medical, USA) were placed under fluoroscopy into the renal pelvis. A 12/14 Fr UAS (Coloplast) was inserted to facilitate stone placement in mid-lower pole calyces, lithotripsy and a 10/12 Fr UAS (Coloplast) for extraction of the stone fragments (0.15 g, respectively ~ 4 mm, rehydrated overnight in 0.9% NaCl). Following a 60-min break to facilitate ureteral dilation, a 7.5 Fr flexible uretero-renoscope (PU3033A, Pusen Medical, China) was used to inspect the calyces. The stones (5–10 stones from human samples in the size range of 1–3 mm) were then positioned using a nitinol grasper (Cook Medical) or water propulsion through the UAS with a syringe. After the stones were introduced, we performed lithotripsy to create heterogenous dust, most of which was ≤ 1.5–2 mm in size. A Thulium fiber laser (Quanta Systems and Coloplast) with a 200 µm fiber was used in a low-energy dusting setting (0.4 Joules, 20 Hz, short pulse). Once lithotripsy was concluded, the urinary tract was occluded with a catheter (IMP Occlusion catheter 6 Ch, Germany) or the UAS to prevent expulsion of fragments. The procedure was concluded when the urinary tract was closed.

*Treatment RIRS (With Magnetic Particle Application)*: The treatment RIRS procedures followed the same steps as the control RIRS till the fragmentation stage. After lithotripsy, we applied 10 mL magnetic particle suspension. After a 1-min incubation, they were extracted using a magnetic probe. The probe was backloaded onto the uretero-renoscope to inspect the kidney and passively retrieve the SRFs. If fragments were still present at the end of the magnetic removal, a second application of magnetic particles (5 mL) and extraction were performed. After completing the treatment, the urinary tract was occluded.

*Computed Tomography Workup*: The CT-examination was conducted by *Radiologisch Nuklearmedizinische Praxis Beck & Kollegen* (Am Stadtpark 5, 15,517 Fürstenwalde/Spree, Germany). Post-euthanization, the kidneys of the animal models were imaged using a Siemens Healthineers SOMATOM-CT scanner with the models in a supine position. We performed a preliminary fast scan to verify model positioning before performing the final images. The resulting data and metadata were exported in DICOM format for analysis, which was carried out using Slicer 5.2.2 (www.slicer.org).

*Histopathological Workup*: All kidneys were evaluated by an independent, accredited veterinarian pathologist (blinded), *Dr. med. Vet. Wolfram Haider, Veterinary Physician for Animal Pathology at the Institute of Animal Pathology* (Schönhauser Straße 62, 13,127 Berlin, Germany). Following nephrectomy, cranial, medial and caudal tissue sections were resected from the renal medulla, pelvis, vein and ureters from each kidney and embedded in paraffin for histopathological examination. Subsequently, a 2 µm-thick section was prepared from each paraffin block, and these sections were stained with hematoxylin and eosin for microscopic examination. Microscopic examination was conducted at 50x, 100x, 200x, and 400 × magnifications to photograph different levels of detail. The samples were investigated for potential toxicity and the occurrence of pathological events was compared between the control and treatment groups as a low sample *t* test. Due to the low sample size in both groups, the difference in occurrences is reported as confidence intervals at 95% confidence with the assumption that the mean difference is zero, since no conclusions about statistical significance can be drawn with high power in this setup. These confidence levels only serve to indicate safety and justify benefit-risk for a clinical investigation.

## Results

RIRS were performed in all kidneys (Fig. 1) and terminated following the study protocol. After complete lithotripsy, fragments were widely disseminated in the collecting systems. Most fragments within the renal pelvis and navigable calyces were magnetized and retrieved using the magnetic system.

**Fig. 2:**
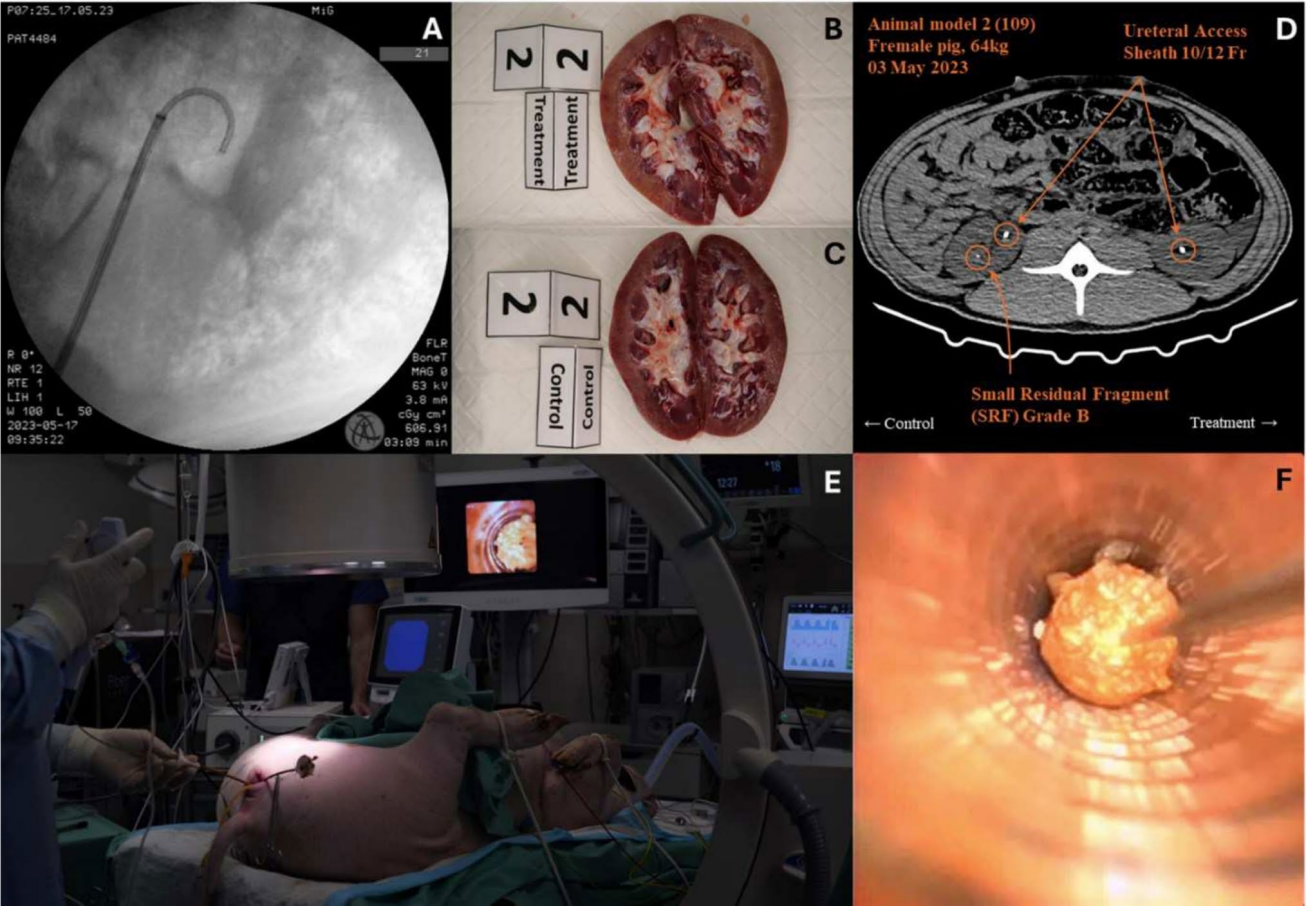
**A**: Positioning of access sheet, **B**: Nephrectomy of a control kidney, **C**: Nephrectomy of a treatment kidney, **D**: CT of animal model, **E**: RIRS and **F**: magnetic extraction

Due to the animal anatomy and coagulation characteristic, we were unable to determine the stone free rate endoscopically. The size of the fragments which were retrieved magnetically were in the range of 0.1 to 4 mm. No adverse events regarding the MP or the magnetic probe were recorded, in particular no signs of toxicity, pyrogenicity or anaphylaxis-associated hypotension or shock (Fig. 2). Urinalysis and hemogram indicated no significant difference in toxicity or effects of the used system between the control and treatment kidneys. None of the porcine models demonstrated signs of post-operative complications pre-euthanization in the acute context.

Histopathological examination revealed damage to the urothelium in both the renal pelvis and ureters in all animals. The size and nature of the damage to the urothelium could only be attributed to laser-lithotripsy and the use of kidney stone baskets during stone implantation. In one animal model, the urothelium was perforated by a damaged guidewire, leading to haematuria, which was controlled within two hours, after which the surgery could proceed. The urothelium throughout the whole circumference was affected by desquamation, which was occasionally focal, but more often occurred in the ureters mainly due to the insertion of the UAS. The differences in occurrence of desquamation (0.159, 95% [−0.235, 0.552]), inflammation (−0.111, 95% [−0.401, 0.179]), haemorrhage (0.005, 95% [−0.094, 0.105]) and the occasional subepithelial oedema (0.074, 95% [0.007, 0.141]) between the control and treatment kidneys were insignificant.

**Fig. 3:**
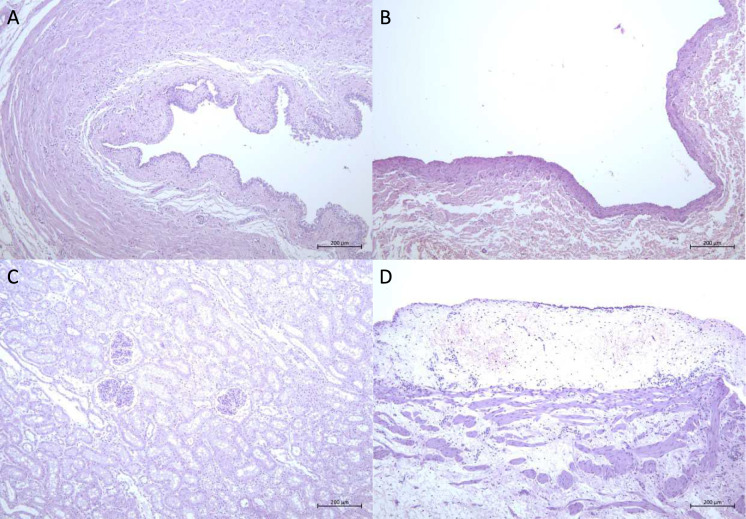
Histopathological staining of **A:** ureter, **B:** vena renalis, **C:** kidney medial and **D:** pelvis medial

MPs show up mainly in low amounts in the renal pelvis, where they occur predominantly free in the lumen. Some particles were visible on the basement membrane of the ureter and renal pelvis, where the epithelium was exposed after desquamation. Small amounts of these particles were also visible in the lumen of the tubules and collecting ducts in the kidneys but did not show acute, pathological effects. The renal papillae were free and revealed no damage. There were occasional blood hemorrhages. They were attributed to anaesthesia and the use of the laser respectively. No physical damage to the kidney tissue could be detected that could have been caused by the treatment. There was an insignificant difference in the occurrence of chronic inflammation in the samples between the groups (0, 95% [−0.226, 0.226]), which is otherwise not an indication of pathological effects caused by the surgery but possible pre-existing infections. None of the renal vein samples showed changes (Table 1 and Fig. 3).

**Table 1:** Histopathological investigation of the kidney

	Control kidneys			Treatment kidneys						
	1-right	2-right	4-right	1-left	2-left	3-left	3-right	4-right	5-left	5-right
Kidney vessels	n.a.d	n.a.d	n.a.d	n.a.d	n.a.d	n.a.d	*Blood congestion in the medulla*	n.a.d	n.a.d	n.a.d
Kidney parenchyma	*Chronic inflammation*	n.a.d	*Chronic inflammation*	*Chronic inflammation*	n.a.d	*Chronic inflammation*	n.a.d	*Chronic inflammation*	n.a.d	*Chronic inflammation, blood congestion*
Inflammation of renal pelvis	Yes, **Haemorrhages**	Yes (focal)	n.a.d	Yes, **Fibrinous exudate, Haemorrhage**	Yes, **Subepithelial oedema, Haemorrhage**	Yes, **Subepithelial oedema, Haemorrhage**	Yes, **Subepithelial oedema,** **Haemorrhages**	Yes **Haemorrhage**	Yes, **Haemorrhage, Subepithelial oedema**	No, **Haemorrhage (mild)**
Urothelium of renal pelvis	**Focal desquamation, Focal vacuolization**	**Extended desquamation, Focal vacuolization**	**Focal desquamation, Moderate vacuolization**	**Focal desquamation, Focal vacuolization**	**Focal desquamation, Focal vacuolization, Fibrinous exudate**	**Focal desquamation, Focal vacuolization**	**Extended desquamation**	**Moderate desquamation, Vacuolization**	**Moderate desquamation**	**Moderate desquamation**
Inflammation of the ureter	Yes	Yes,** Haemorrhage, Fibrinous exudate**	Yes (focal), **Haemorrhage, Subepithelial oedema**	Yes	Yes	Yes (focal)	Yes, **Haemorrhages**	Yes, **Haemorrhages, Fibrinous exudate**	Yes	Yes (focal)
Urothelium of the ureter	**Nearly complete desquamation**	**Extended desquamation**	**Focal desquamation, Moderate to severe vacuolization**	**Extended desquamation**	**Focal desquamation**	**Extended desquamation, Extended vacuolization**	** ~ 30% desquamated, Extended vacuolization**	**Moderate desquamation**	**Focal desquamation, Moderate vacuolization**	** ~ 30–50 percent desquamated**
Vena renalis	n.a.d	n.a.d	n.a.d	n.a.d	n.a.d	n.a.d	n.a.d	n.a.d	n.a.d	n.a.d

All acute histopathological changes are signalled in bold letters. Each tissue type of each kidney had caudal, medial and cranial sections resected. This table summarizes caudal, medial and cranial results into one qualitative description

(‘n.a.d.’ stands for “nothing abnormal detected”). *CT*: Reconstructed images from the high-dose CT imaging of all five porcine models with 0.7 mm section thickness revealed the SRFs in both the control and treatment kidneys. 1/7 kidneys were Grade I whereas 6/7 kidneys were Grade II according to the Clavien-Dindo classification [28, 29]. The kindeys showed high stone-free ratesaccording to the guidelines [4]. A further quantification was not possible since the extractable fragments could not be differentiated from the fragments that tightly adhered to the clots on the urothelium

## Discussion

Urolithiasis has been a long-standing medical challenge, and despite the significant advancements in medical technology, the prevalence and incidence of kidney stones have continued to rise in all populations groups. Recent research has challenged the use of the term CIRFs among urologists [2]. Emerging evidence suggests that SRFs ≤ 2 mm (RF ≤ 4 mm) have a substantial impact on the future well-being of patients [1, 2, 30, 31]. Consequently, the primary objective of RIRS should be the prompt and complete clearance of stones [9, 31]. Current lithotripsy techniques often leave the surgeons with the challenge of extracting large SRF volumes for medium and large renal stones (> 10 mm). Retrieving these fragments time-consuming and laborious, frequently resulting in incomplete removal. Moreover, existing devices struggle to grasp SRFs measuring less than 2 mm, which can serve as nuclei for future stone regrowth and related complications [2]. To address this challenge, magnetic extraction offers a potential solution [21, 32]. The presented data highlights the first in vivo application of this innovative magnetic particle system for the retrieval of SRFs and successfully demonstrated the practicality of this magnetic system in RIRS. This approach has the potential to significantly reduce procedure time by allowing the handling of multiple fragments up to 3—4 mm globally in the kidney, including very small pop-dusted fragments, compared to the laborious process of sequentially collecting numerous hyperlocalized fragments. The extraction time for each kidney was below 30 min in order to comply with guidelines to keep treatments within 90 min and to be comparable with other extraction techniques such as basketing [4]. Importantly, this procedure has shown promise in terms of safety, tolerance, and atraumatic ability for the acute treatment. None of the animals displayed any signs of toxic or anaphylactic reactions during surgery, and histopathological assessments reported no injuries due to the devices compared to the reference kidney (Table 1 and Fig. 2). It is worth noting that, due to the significantly faster metabolism of porcine models compared to humans, any histopathological abnormalities become readily apparent [33, 34]. Hemorrages have been visible which can be partially associated with tissue injury. All vein samples were free of particles, demonstrating that the particles, in the acute context, did not turn intravascular. However, it is essential to acknowledge the limitations associated with the porcine animal model used in this study. Porcine coagulation and anatomy features present unfavourable aspects, such as distinct kinking of ureters and ureteropelvic junction or very fast clotting, as noted by others [35]. Porcine blood is in a hypercoagulable state and the intrinsic coagulation system is hyperactive, which meant that stone fragments post-lithotripsy embedded themselves strongly to the clotting sites [26]. This hindered their magnetic extraction and were therefore indistinguishable from extractable SRFs post-surgery during the CT-evaluation. A quantification endoscopically between the control and treatment group was also not possible.

## Conclusions

This porcine in vivo study introduced a novel technology using magnetic extraction to address the challenges associated with kidney stone removal, especially the SRFs generated during laser-lithotripsy irrespective of settings (heterogenous SRFs). The application of magnetic particles proved to be safe and well-tolerated for acute treatment, and our findings affirmed the system’s feasibility and acute safety in vivo. It also demonstrated that SRFs can be effectively managed through passive, magnetic, endoscopic attraction of multiple fragments. While the preclinical evaluation of magnetic particles is promising, further research is necessary. Clinical trials involving human patients are required to validate the effectiveness and safety of magnetic particles in real-world scenarios. Sub-chronic studies are also essential to evaluate the risk of potential particle residues.

## Data Availability

No datasets were generated or analysed during the current study.
